# What Happens to Patients on Antiretroviral Therapy Who Transfer Out to Another Facility?

**DOI:** 10.1371/journal.pone.0002065

**Published:** 2008-04-30

**Authors:** Joseph Kwong-Leung Yu, Teck-Siang Tok, Jih-Jin Tsai, Wu-Shou Chang, Rose K. Dzimadzi, Ping-Hsiang Yen, Simon D. Makombe, Amon Nkhata, Erik J. Schouten, Kelita Kamoto, Anthony D. Harries

**Affiliations:** 1 Taiwan Medical Mission in Malawi, Mzuzu Central Hospital, Mzuzu, Malawi; 2 International Medical Cooperation and Development Center, Pingtung Christian Hospital, Pingtung, Taiwan; 3 College of Medicine, Kaohsiung Medical University Division of Infectious Disease, Kaohsiung Medical University Hospital, Kaohsiung, Taiwan; 4 Bureau of International Cooperation, Department of Health, Taipei, Taiwan; 5 Office of the Hospital Director, Mzuzu Central Hospital, Ministry of Health, Mzuzu, Malawi; 6 HIV Unit, Ministry of Health, Lilongwe, Malawi; 7 Management Sciences for Health, Lilongwe, Malawi; 8 Family Health International, Lilongwe, Malawi; 9 London School of Hygiene and Tropical Medicine, London, United Kingdom; Instituto de Pesquisa Clinica Evandro Chagas, FIOCRUZ, Brazil

## Abstract

**Background:**

Long term retention of patients on antiretroviral therapy (ART) in Africa's rapidly expanding programmes is said to be 60% at 2 years. Many reports from African ART programmes make little mention of patients who are transferred out to another facility, yet Malawi's national figures show a transfer out of 9%. There is no published information about what happens to patients who transfer-out, but this is important because if they transfer-in and stay alive in these other facilities then national retention figures will be better than previously reported.

**Methodology/Principal Findings:**

Of all patients started on ART over a three year period in Mzuzu Central Hospital, North Region, Malawi, those who transferred out were identified from the ART register and master cards. Clinic staff attempted to trace these patients to determine whether they had transferred in to a new ART facility and their outcome status. There were 805 patients (19% of the total cohort) who transferred out, of whom 737 (92%) were traced as having transferred in to a new ART facility, with a median time of 1.3 months between transferring-out and transferring-in. Survival probability was superior and deaths were lower in the transfer-out patients compared with those who did not transfer.

**Conclusion/Significance:**

In Mzuzu Central Hospital, patients who transfer-out constitute a large proportion of patients not retained on ART at their original clinic of registration. Good documentation of transfer-outs and transfer-ins are needed to keep track of national outcomes. Furthermore, the current practice of regarding transfer-outs as being double counted in national cohorts and subtracting this number from the total national registrations to get the number of new patients started on ART is correct.

## Introduction

Malawi, a small resource-poor country in Southern Africa, has been making good progress with scaling up antiretroviral therapy (ART): by June 2007, just over 110,000 patients had started free treatment from 109 public sector facilities [1]. Treatment outcomes are monitored every quarter, with patients classified as alive and on treatment, dead, stopped treatment, transferred to another facility or “lost to follow-up”. The category of transfer-out is used in both the ART programme and national TB programme to indicate patients who actively seek a transfer to another treatment facility. As new sites are continuously being accredited for ART delivery, many patients who are started on therapy in a distant hospital obviously prefer to continue with therapy from a facility nearer to home and therefore transfer. By June 2006, there were 9,862 patients (9% of the total cohort) who had been recorded as transfer-out in the ART registers from around the country [1].

There is recent concern about the poor retention on therapy in ART programmes in sub-Saharan Africa [2], [3]. Reports on treatment outcomes from individual ART clinics in sub-Saharan Africa often raise the problem of loss to follow-up [4]–[9]. However, surprisingly little mention is made of transfer-outs, with one study from Botswana mentioning a transfer-out rate of 5.2% [4], and one from South Africa mentioning this as a small component of loss to follow-up [5]. We know from the Malawi national quarterly ART reports that transfer-outs are a common cause of patients no longer being retained on therapy in their original ART clinic. However, we have no hard data on whether these patients transfer-in to another facility and we do not know how they fare in terms of treatment outcomes once registered in the new facility. We therefore conducted a study in the Northern Region of Malawi to investigate this issue, and to determine what had happened to patients who transferred out from Mzuzu Central Hospital to other facilities.

## Materials and Methods

### Background

Details of how ART in Malawi is delivered and monitored have previously been described [10]. When patients start ART, their details are entered into ART patient master cards and an ART register, and patients are given a unique ART identification number. Every month patients come for clinic reviews, at which time their outcome status is entered into the master card and they are given another supply of ART drugs for a month. If a patient wishes to transfer to another facility, the name of the new facility to which the patient will move is indicated in the ART register and the treatment outcome is changed to transfer-out. The patient is given his/her ART master card, a transfer out letter and a month's supply of drugs to take to the new facility. A copy of the original master card is made and kept in the usual numerical order in the arch-back files in the original clinic [10]. Thus, ART staff and staff from supervising teams can see who has transferred out from each clinic by going through the ART patient master cards and the register.

At the new facility, the patient is registered with a new ART identification number related to that facility. The patient hands over his/her master card from the previous site, and on this master card the ART identification number is changed to the new number. This master card is then filed in the usual way and used until the end of the year, at which time a new master card is issued to the patient. In both the ART register and the ART master card it is clearly written that the patient is a “transfer-in”.

### Data collection and Analysis

The master cards and the ART register of all new patients registered at Mzuzu Central Hospital, Northern Region, Malawi, between June 2004 and December 2006 were reviewed, and a record was made of those who had transferred out to another facility. For patients who had transferred out within the Northern Region, active visits were made to all the ART clinics in that region to determine whether patients had in fact transferred-in, the date of the transfer-in and their latest treatment outcome with the date. Treatment outcomes of patients transferring in were censored on March 31^st^ 2007. Patients alive and on ART on March 31^st^, 2007, were recorded as such. However, those who had died, were lost to follow-up, stopped therapy or transferred-out again before this date were recorded as such with the date of this outcome. For patients transferring to the Central or Southern Region, active follow-up was confined to making a phone call to the new site to try and obtain data similar to that obtained during Northern Region site visits.

Categorical variables between patients who transferred out and who did not transfer out were analyzed and compared using the chi-squared test with odds ratios (OR) and the chi-squared test for trend. The probability of survival between patients who transferred out and were traced and patients who did not transfer-out was estimated using the Kaplan-Meier method, and comparisons made using the Cox-Mantel (Log rank test). The level of significance was set at P = 0.05 or less, and 95% confidence intervals (CI) were used throughout. Data analysis was carried out using SAS system for Windows (Version 8.01).

### Ethical Approval

The Malawi National Health Science Research Committee provides general oversight and approval for the collection and use of routine programmatic data for monitoring and evaluation, as was the case with this study.

## Results

There were 4175 patients registered for ART, of whom 805 (19%) had transferred out to another facility, 65 patients transferring to the Central and Southern Regions and 740 to the Northern Region. Altogether, 737 (92%) of these patients were traced. Tracing was successful in 16 (25%) of 65 patients who transferred to the Central and South region and 721 (98%) 740 patients who transferred to the Northern Region.

**Figure 1:**
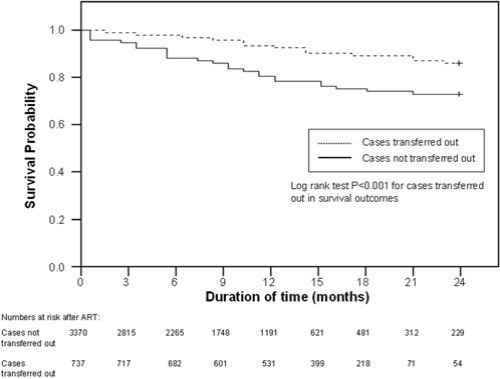
Probability of Survival in Patients on ART who Transfer Out and who do not Transfer Out.

Characteristics and treatment outcome status of patients who transferred out and were traced compared with those who did not transfer out are shown in **Table 1**. There was a trend for those transferring out to have less advanced clinical stage of disease (WHO Clinical Stage 1,2 or 3 together) compared with those who did not transfer out. The proportion of transfer-out patients who died was significantly less than those who did not transfer out [OR 0.4, 95% CI 0.3–06], and of those transfer-out patients who did die, there was a significant trend towards later deaths compared with those who did not transfer out. There was a significantly higher survival probability in patients transferring out and traced compared with those who did not (**Figure 1**).

**Table 1:** Characteristics and treatment outcomes of patients who transferred out and those who did not transfer out from Mzuzu Central Hospital, Malawi, between June 2004 and December 2006.

	Patients transferring out	Patients not transferring out	P value
Number of patients started on ART	805	3370	
Female patients	491 (61%)	1977 (59%)	NS
Children (aged<15 years)	81 (10%)	396 (12%)	NS
Median age in years	36	37	NS
Indication for ART:			
WHO Stage 1 or 2 with low CD4 count	56 (7%)	530 (16%)	p = 0.016^b^
WHO Clinical Stage 3	620 (77%)	2186 (65%)	
WHO Clinical Stage 4	129 (16%)	654 (19%)	
Patients transferring out and not traced	68		
Patients transferring out and traced	737		
Median time (range) in months between transfer out and transfer in those traced	1.3 (0.03–3.53)		
Treatment outcome status^a^			
Alive and on ART	634 (86%)	2826 (84%)	NS
Dead	40 (5%)	423 (12.5%)	p<0.001
Lost to follow-up	22 (4%)	120 (3.5%)	NS
Stopped treatment	0	1	NS
Transferred out again	41 (5%)	Not applicable	
Month of death after start of ART in the original or the new facility for those who had transferred out:			
Month 1	0	180 (42.5%)	
Month 2	4 (10%)	74 (17.5%)	p = <0.001^c^
Month 3	5 (12.5%)	43 (10%)	
After month 3	31 (77.5%)	126 (30%)	

a treatment outcomes censored on March 31^st^, 2007, and for those who transferred out these refer to those who were traced.

b chi-squared test for trend = 5.8.

c chi-squared test for trend = 42.3.

WHO = World Health Organization; ART = antiretroviral therapy.

## Discussion

This study shows first of all that almost one fifth of patients transferred out from a central hospital institution over a 30-month period as new ART sites were set up in the country and started to deliver therapy closer to patients' homes. For geographical reasons more patients transferred out to new sites in the Northern Region compared to the other 2 regions in the country.

Second, we were able to document that over 90% of these patients transferred in to a new facility. Tracing was more successful in the Northern Region where active follow-up was conducted to sites compared with the other 2 regions of the country where only telephone calls could be made. The median time for the transfer process was just over 1 month, which suggests that patients who usually move with a new 1-month's supply of drugs probably do not experience drug interruptions during the transfer process, an important factor in reducing the development of drug resistance. The reasons for not finding what happened to some transfer out patients are speculative. These patients may have transferred to a different site altogether either within or outside of Malawi, they may have used a different name to transfer to another site, or they may have died or decided to stop therapy.

Third, the probability of survival in patients transferring out was better than those who remained at the central hospital, suggesting that transfers occur after patients have stabilised on therapy and after the first three months when a large proportion of ART deaths occur [5], [9].

There are two important lessons from this study. First, two recent published reports have emphasised that there is poor retention on therapy in Africa's ART programmes, citing loss to follow-up and death as the principal reasons for attrition [2], [3]. We feel that these reports are potentially misleading, and that one of the important reasons for poor ART clinic retention is the transfer out of patients who move to another site yet continue to successfully take ART. In Malawi's well organised tuberculosis programme, there were poor quality data on transfers [11], and in Malawi's national ART programme we are well aware that patients may transfer-out without informing the original clinic, and these patients will be counted as “losses to follow-up” unless active tracing is conducted [12]. Second, in Malawi we have thought for some time that patients who transfer out may be double counted, in their original ART site as a transfer-out and in the new site with a new number as alive and on therapy. To allow for this at national level, when we count the number of new patients starting on therapy we subtract the transfer-outs from the total national registrations. The current study validates this approach, as over 90% of patients who transfer-out are indeed registered at a new site and the majority of those registered are subsequently found alive and on ART.
